# Frailty Screening and Management for Older Australians in General Practice: Mixed Methods Evaluation

**DOI:** 10.2196/79681

**Published:** 2026-03-02

**Authors:** Jennifer R Job, Caroline Nicholson, Ruby Strauss, Debra Clark, Anita Pelecanos, Claire Jackson

**Affiliations:** 1 Centre for Health System Reform and Integration, Mater Research-The University of Queensland, Level 8, Health Sciences Building, RBWH, Brisbane, 4006, Australia, 61 7 3365 5379; 2 Aged Care, Sydney North Health Network, Sydney, Australia; 3 Statistics Unit, QIMR Berghofer Medical Research Institute, Brisbane, Australia; 4 General Practice Clinical Unit, The University of Queensland, Brisbane, Australia

**Keywords:** FRAIL scale, implementation, older adults, frailty screening, primary care

## Abstract

**Background:**

Frailty increases with age and is associated with increased vulnerability to adverse health outcomes. International guidelines recommend screening for frailty in primary care; however, this is not routine practice in Australia. Once identified, frailty progression has the potential to be halted or reversed with early intervention. The FRAIL (Fatigue, Resistance, Ambulation, Illnesses, Loss of weight) Scale Tool, a simple and validated screening and management tool, offers a feasible approach for integration into the Australian health assessment for those aged 75 years and older (75+HA), which can be performed annually by primary care providers.

**Objective:**

This study explores the rates of frailty, resources required to support management, and the determinants of implementing frailty screening and providing management for older Australians at the 75+HA.

**Methods:**

A mixed methods evaluation was conducted in 24 general practices across 2 Australian Primary Health Network regions, Sydney North and Brisbane South. The FRAIL Scale Tool was implemented during the 75+ health assessment, and data were collected on FRAIL Scale scores, hospitalization rates, recommended frailty interventions, and barriers to frailty management. Practice staff perceptions of the long-term sustainment of the FRAIL Scale Tool were assessed using the Provider Report of Sustainment Scale. Semistructured qualitative interviews were conducted with practice staff and patients, exploring barriers and enablers to implementing frailty screening and management. Guided by the Consolidated Framework for Implementation Research, transcripts were coded and themes developed.

**Results:**

Of the 1484 patients aged ≥75 years who were screened, 223 (15%) patients were frail, 616 (41.5%) patients were prefrail, and 645 (43.5%) patients were robust. People who were frail were more likely to be female, older, and have more prescribed medications. Of those screened as frail, 23 (11%) had a nonelective hospitalization in the 3 months prior to screening compared with 28 (5%) who screened as prefrail and 5 (1%) who screened as robust (*P*=.012). Management recommendations commonly included medication reviews, aged care packages, assessment for depression, and exercise programs. Barriers identified to accessing interventions included health, transport, cost, and time. Survey and qualitative findings highlighted that the FRAIL Scale Tool was easy to use, integrated well into existing workflows as part of the 75+HA, and sustained use would be supported by software integration. Patients valued the assessment and tailored health support offered by trusted primary care providers.

**Conclusions:**

Incorporating the FRAIL Scale Tool into the annual health assessment for people aged 75 years and older provides a funded opportunity for addressing frailty in general practice. Patients and staff value the Tool’s simplicity and the opportunity to raise awareness and manage frailty proactively. Incorporating the Tool into practice software systems would enhance adoption. Broader implementation research in diverse settings and with Aboriginal and Torres Strait Islander populations is needed to improve frailty prevention and management.

## Introduction

Worldwide, the population is aging with an increasing proportion of people aged 60 years and older, and the number of people aged 80 years or older is expected to triple between 2020 and 2050 [1]. In response, health care systems and models of care must focus on the health needs and priorities of older people. Frailty and its precursor, prefrailty, affect more than half of the adults older than 50 years worldwide [2]. Characterized by physiological decline and increased vulnerability to stressors, frailty is a dynamic state associated with an increased risk of adverse health outcomes [3]. While not an inevitable part of aging, the risk of developing frailty increases with advancing age, with the prevalence of frailty and prefrailty climbing to 31% and 52% respectively, in people older than 80 years [2]. With the gradual loss of muscle mass and decline in physical condition, frailty is associated with falls and acute illnesses. Hospitalization or prolonged recovery can contribute to further physical deconditioning, with people experiencing frailty often requiring substantial support and resources to return to their prior health state or prevent further deterioration [4]. Importantly, causes of frailty can be managed, and in some cases reversed, highlighting the importance of early identification of older people who are living with prefrailty [5].

International guidelines highlight the opportunity for frailty prevention and early intervention in primary care [6]. This can be challenging as prefrailty develops subtly and may go unnoticed without targeted examination. While screening of older adults in primary care has been recommended for early identification of prefrailty to initiate preventative management strategies [7], screening is not routinely recorded and tracked in practice in Australian primary care [8]. This presents a missed opportunity as validated screening tools have been found to assist health care professionals with identifying risk of frailty and monitoring any changes in health status [6].

**Figure 1.**
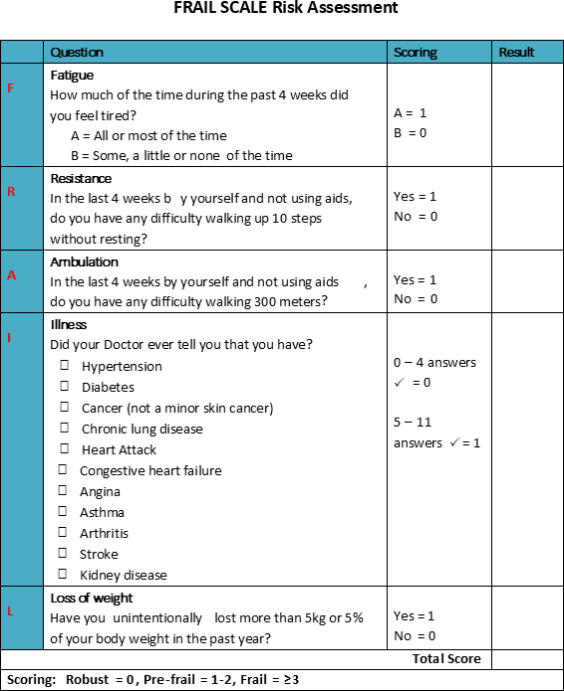
The FRAIL Scale. FRAIL: Fatigue, Resistance, Ambulation, Illnesses, Loss of weight.

The FRAIL (Fatigue, Resistance, Ambulation, Illnesses, Loss of weight) Scale (Figure 1) has been validated [9,10] as a screening tool for community-dwelling adults. A recent pilot study found the Tool was feasible for general practitioners (GPs) and practice nurses to administer in a busy clinical setting and appropriate for screening older people at the time of routine appointments [11]. It is based on the phenotype of frailty elements of Fried et al [12]: exhaustion (fatigue), weight loss, measured grip strength and walking speed, and low energy expenditure. The FRAIL Scale includes 5 simple questions about fatigue, resistance, ambulation, illnesses, and loss of weight to identify risk of frailty [9]. FRAIL Scale scores range from 0 to 5 (with 1 point for each component) to give one of three outcomes: (1) frail (3 or more components present), (2) prefrail (1 to 2 components present), or (3) robust (0 components present).

**Figure 2.**
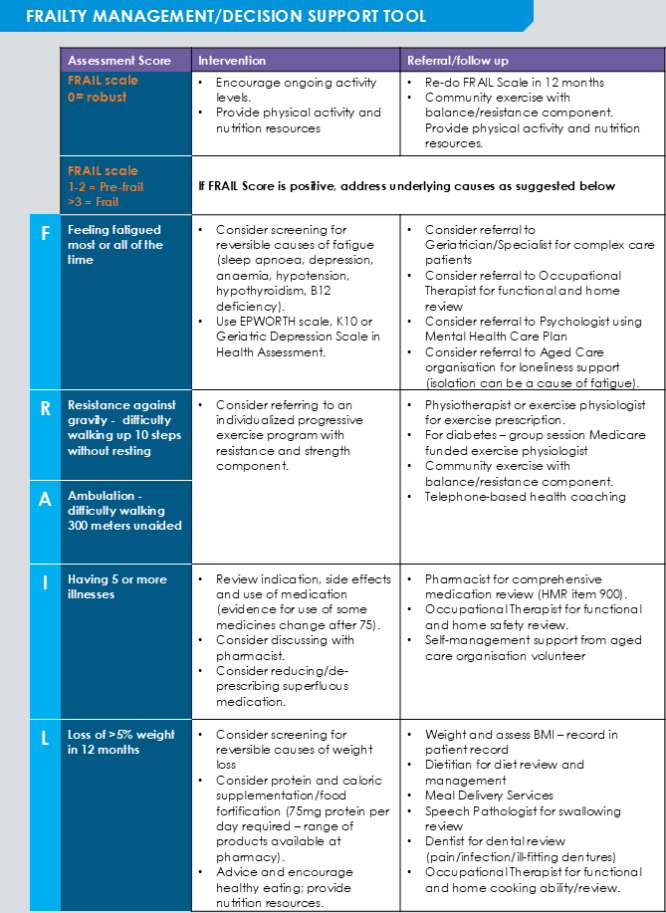
The Frailty Management Decision Tool (The FRAIL Scale Tool).

Once identified, frailty progression has the potential to be halted or reversed with proper management [5]. Clinical guidelines suggest a multifaceted management approach to address physical function, nutrition, polypharmacy, and fatigue [6,13,14]. However, according to health professionals, the management of frailty can be hindered by a lack of defined protocols and care pathways to guide evidence-based intervention in practice [15,16]. To address this issue, the bespoke Frailty Management/Decision Tool (the Tool) was developed by Sydney North Health Network (SNHN) and Northern Sydney Local Health District for primary care staff to assess and track FRAIL Scale scores and provide associated management suggestions and interventions recommended within the Tool (Figure 2). The tool was successfully piloted by general practices in Sydney North and Brisbane South, with 80% (860/1071) of those screened identified as frail or prefrail [11]. Staff feedback highlighted that the Tool was patient-focused, easy to use, and a formalized assessment for risk of frailty. To fit with practice workflows, this pilot research supported screening for frailty risk in conjunction with the annual Medicare-funded preventive health assessment for those aged 75 years and older (75+HA), which is conducted with more than 600,000 older Australians in general practice annually [17]. While the 75+HA assesses falls risk, vision, hearing, current weight, and cognitive assessment, it does not currently include a formalized frailty assessment.

This study aims to determine:

1. The rates of risk of frailty/frailty in the general practice population aged 75 years and older when screened at the time of the 75+HA.
2. The resources required to support those identified as frail or at risk of frailty.
3. Barriers and enablers to implementing frailty screening and management for community-dwelling older Australians in primary care.

## Methods

### Design

A convergent mixed methods study design was used. FRAIL Scale scores, recommended resources for frailty management, and barriers to accessing these were tracked prospectively by general practice staff. Semistructured qualitative interviews were conducted with practice staff and patients to understand (1) the determinants of implementing frailty screening and (2) the barriers and enablers to frailty management in primary care. Interview questions and analysis were guided by the Consolidated Framework for Implementation Research (CFIR) [18]. CFIR provides a framework for assessing determinants of implementation at multiple contextual domain levels: inner and outer setting, intervention, individual, and implementation process.

### Researcher Positionality

DC is a Primary Health Network (PHN) stakeholder who worked with Sydney North primary care centers to introduce the FRAIL Scale tool. AP contributed as a biostatistician, and the remainder of the research team has worked clinically with older persons (JJ: Dietitian, CN: Physiotherapist, RS: Sleep Technician, CJ: GP). All have extensive experience implementing health services in the primary care setting and are acutely aware of the time and workforce challenges faced by primary care.

### Ethical Considerations

Ethics was approved by The University of Queensland HREC (Project IDs: 2021/HE001441 and 2021/HE002398), and informed consent was obtained from participants. Participant data was protected by unique study codes and secure servers with limited access to ensure confidentiality, and only deidentified data was shared with the researchers. General practice incentive payments supported the general practice staff time for the data collection (70 frailty screens/practice at Aus $40 [US $26.652]/assessment for new practices). Participants were reimbursed for their time for a semistructured interview at Aus $120 (US $79.956)/interview for practice staff and Aus $50 (US $33.315) for patients.

### Recruitment

As previously described, the research team partnered with 2 PHNs—SNHN and Brisbane South Primary Health Network (BSPHN)—to pilot [11] and more broadly implement the Frailty Management Decision tool. PHNs are not-for-profit organizations funded by the Australian Government to improve access to primary care services for patients, particularly those at risk of poor health outcomes, and coordination of care.

SNHN is a predominantly metropolitan region that is ranked the least socioeconomically disadvantaged PHN in Australia. In contrast, BSPHN is a diverse region that includes metropolitan, rural, and remote island locations, and 2.8% of residents are Aboriginal or Torres Strait Islander. There are 292 general practices in SNHN [19] and 336 in BSPHN [20], with people aged ≥65 years making up 17.3% of SNHN residents and 14.2% of BSPHN residents.

Practices that participated in the pilot program between April 2021 and June 2023 were invited to join this study. Additional practices were recruited between July 2023 and February 2024 via a PHN practice newsletter, expressions of interest, and PHN/researcher practice networks. The Tool was implemented by 24 enrolled practices (14 SNHN, 10 BSPHN).

### Tool Implementation

Patients were screened using the FRAIL Scale tool during the 75+HA. GPs, nurses, and other practice staff completing 75+HA were encouraged to use an optimized health assessment template incorporating the FRAIL Scale questions (Multimedia Appendix 1) or add the FRAIL Scale to their existing practice templates. The frailty score was calculated, and practices referred to the associated management suggestions and interventions recommended within the Tool (Figure 2). Practice staff could download the results of the questionnaire and save them to the patient’s medical record.

Training for the practices included (1) an in-person, phone, or web-based video session outlining the aims of the study, how to complete data collection, and use of the FRAIL Scale tool, and (2) check-in visits, or phone/video calls, and emails with the project researcher. Data collection commenced in July 2023 and was completed in January 2025.

### Data Collection

#### Implementation Evaluation

During the study, practice staff conducted FRAIL Scale screening with patients aged 75 years and older and collected deidentified data via a REDCap (Research Electronic Data Capture; Vanderbilt University) data collection tool developed for the research. Data collected included date of screening, time to complete the screen, patient characteristics (age, BMI, sex, and Aboriginal or Torres Strait Islander background); whether the patient had been hospitalized (planned or unplanned) or attended the emergency department in the previous 3 months; current medication number; patient FRAIL Scale scores; suggested management/referral options; and any barriers identified by patients or caregivers for accessing suggested management/referral options from a picklist (cost, time, transport, or health) or a free text option.

Practices provided data on the active practice population and the total number of GPs and nurses working in the practice. Practices were classified on location (ie, metropolitan or regional) using the Modified Monash Model [21].

#### Staff Survey

Staff perceptions of the long-term sustainment (continued use) of the FRAIL Scale were assessed using the Provider Report of Sustainment Scale (PRESS), a pragmatic, reliable, valid 3-item survey [22]. The 3 items were rated on a scale from 0 (not at all) to 4 (to a very great extent), with the mean score calculated and higher scores indicating more sustainment. An additional free-text option was included: “Do you have any other feedback regarding the implementation of the FRAIL Scale tool in your practice?”

#### Qualitative

Qualitative interviews were conducted with (1) practice staff who had used the FRAIL Scale tool during implementation, and (2) patients who had been assessed for risk of frailty to understand determinants (barriers and enablers) to (1) implementing the FRAIL Scale tool and (2) risk of frailty management in primary care.

Staff conducting the FRAIL Scale tool assessments during the study were invited to be interviewed, and practice staff recruited potential patients for interview at the time of the 75+HA. Invitations were emailed or mailed to staff and patient participants, with information describing the rationale for the research provided via an information sheet. Participants were sent the interview questions in advance, allowing them time to consider their viewpoints. Interviews were semistructured and informed by the CFIR [18,23]. The relevance of each CFIR determinant to the implementation of the FRAIL Scale tool was rated by the research team members (CN, JJ, and DC), and those with the highest rating were used to determine interview questions [24]. Interview questions Multimedia Appendix 2) were developed using the Interview Guide Tool available via the CFIR website [25] and with a focus on patient views on the FRAIL Scale assessment tool, barriers to accessing management options and resources, and preferences for suitable options and resources. All interviews were conducted via telephone by an implementation scientist with extensive experience in qualitative research (JJ). As an alternative to data saturation, the process of Malterud et al [26] was used to inform the sample size. Sample size was determined to be met once the purpose and goals of the analysis were achieved, with information power considered strong, since the interviewer was experienced, the research questions were specific, and the interview guide was informed by theory [26]. The interviewer recorded notes during and after interviews to identify barriers and enablers to implementing screening and management for risk of frailty/frailty (JJ). Interviews were audio-recorded, transcribed verbatim using Microsoft Word, cross-checked against the recording (JJ), and anonymized.

### Data Analysis

#### FRAIL Scale

Data were summarized using median (IQR) for continuous nonnormally distributed variables and frequency (percent) for categorical variables. The associations between frailty (frail, prefrail, and robust) and patient demographics were explored using chi-square tests for categorical characteristics and Mann-Whitney *U* tests for continuous nonnormally distributed characteristics. Statistical significance was indicated at a *P* value of <.05 (2-sided). These analyses were performed in STATA (version 18; StataCorp LLC) and IBM SPSS Statistics (version 29.0.0.0 241).

#### PRESS Survey

Survey data were presented using descriptive statistics (median and IQR) and tabulated with free-text responses.

#### Qualitative interviews

A deductive, thematic analysis was used to understand barriers and enablers to implementation of frailty screening and management in general practice with a view to guiding ongoing implementation [27]. Interview transcripts were coded by 2 researchers (JJ and RS), using a deductive process guided by CFIR [18], and themes were developed (JJ and RS). The aim of having 2 coders was not to reach consensus, but to develop richer and more complex insights into the data. As every participant has their own experience and view of frailty screening and management, member checking of results was not conducted [28].

The qualitative feedback was used to explore, in greater depth, the (1) results of the PRESS survey on sustainment of the Tool and (2) data collected by GPs and nurses from patients regarding the barriers they identified to accessing frailty management resources [29].

## Results

### Overview

The characteristics of the Sydney North and Brisbane South practices that implemented the FRAIL Scale tool at the time of the 75+HA are presented in Table 1. All practices were in a metropolitan region (MMM1) [21]. Screening was conducted by 61 staff (57 nurses, 3 GPs, and 1 nondispensing pharmacist) from the practices who used the FRAIL Scale tool.

**Table 1.** Practice demographics.

	FRAIL^a^ Scale implementation practices, median (IQR)		
	All (n=23)	BSPHN^b^ (n=9)^c^	SNHN^d^ (n=14)
Practice patient population	6207.0 (3906.0‐8477.0)	7194 (5623.0‐1999.5)	4167.5 (2945.8‐6779.3)
Number of active patients aged ≥75 years	429.0 (209-878)	461.0 (429.0‐1272.0)	348.0 (176.5‐597.5)
Number of GPs^e^ in the practice	7.0 (5.0‐11.0)	8.0 (5.5‐11.5)	6.5 (4.8‐11.3)
Number of practice nurses	2.0 (2.0‐4.0)	2.0 (1.4‐4.3)	2.5 (2.0‐4.0)

a FRAIL: Fatigue, Resistance, Ambulation, Illnesses, Loss of weight.

b BSPHN: Brisbane South Primary Health Network.

c Missing data for one BSPHN practice.

d SNHN: Sydney North Health Network.

e GP: general practitioner.

### Frailty Screening Results

Practice staff screened 1484 patients for risk of frailty at the time of the 75+HA (Table 2). The median time taken by staff to complete the FRAIL Scale tool was 3.9 minutes (IQR 2.2‐7.6 minutes, n=1458).

**Table 2.** Patient demographics for Brisbane South and Sydney North primary health networks.

	Total (n=1484)	Brisbane South (n=588)	Sydney North (n=896)
Patient sex, n (%)			
Male	650 (43.8)	256 (43.5)	394 (44.0)
Female	834 (56.2)	332 (56.5)	502 (56.0)
Age in years at screen (years), median (IQR)	80 (77-85)	79 (77-84)	81 (77-85)
BMI (kg/m^2^), median (IQR)^a^	26.8 (23.7-30)	27.6 (24.4-30.8)	26.2 (23.3-29.6)
Aboriginal or Torres Strait Islander, n (%)^b^	7 (0)	2 (0)	5 (1)
Prescribed medication count, median (IQR)^c^	5 (3-8)	5 (3-8)	5 (3-7)
Frail score, median (IQR)	1 (0-2)	1 (0-2)	1 (0-2)
Nonelective hospitalization in prior 3 months, n (%)^d^	56 (4)	20 (4)	36 (4)
Emergency department attendance in prior 3 months, n (%)^e^	90 (6)	38 (6)	52 (6)

a Missing data, n=271

b Missing data, n=40.

c Missing data, n=5.

d Missing data, n=26.

e Missing data, n=5.

At the time of screening, 43.5% (n=645) of all patients (n=1484) were classified as robust (score 0), 41.5% (n=616) as prefrail (scores 1‐2), and 15.0% (n=223) as frail (scores 3‐5) (Table 3). People who were frail were more likely to be female, older, have more prescribed medications, and to have had a nonelective hospitalization in the prior 3 months. Although those who were robust had a lower BMI than those who were prefrail or frail (statistically significant), the median BMI did not differ greatly between the frail groups.

**Table 3.** Patient characteristics by FRAIL (Fatigue, Resistance, Ambulation, Illnesses, Loss of weight) Scale score.

	Frail score			*P* value
	Robust (0) (N=645)	Prefrail (1-2) (N=616)	Frail (≥3) (N=223)	
Patient sex, n (%)				.045
Male	299 (46.4)	269 (43.7)	82 (37)	
Female	346 (53.6)	347 (56.3)	141 (63.2)	
Age in years at screen (years), median (IQR)	79 (77-83)	80 (77-85)	84 (79-88)	<.001
BMI (kg/m^2^), median (IQR)^a^	26.2 (23.5-29.1)	27.3 (24-30.8)	27.1 (23.5-31.2)	<.001
Prescribed medication count, median (IQR)^b^	4 (2-5)	6 (4-9)	8 (5-11)	<.001
Nonelective hospitalization in prior 3 months, n (%)^c^	5 (1)	28 (5)	23 (11)	.01
Emergency department in prior 3 months, n (%)^d^	20 (3)	42 (7)	28 (13)	.82

a Missing data, n=271.

b Missing data, n=5.

c Missing data, n=26.

d Missing data, n=5.

The predominant frailty management or resources suggested are outlined in Table 4, with GP medication reviews most frequently recommended (17.3%).

**Table 4.** Management recommendations and resources (10 most frequent for prefrail and frail patients who had recommendations made of the 1484 screened).

Recommendation or resource	Values, n (%)
GP^a^ medication review (medication indications/side effects/usage)	256 (17.3)
My Aged Care packages	194 (13.1)
Use depression assessment in 75+HA^b^ tool	141 (9.5)
Activity program/ exercise classes	108 (7.3)
Fatigue - screen for sleep apnea, depression, anemia, hypotension, hypothyroidism, B12 deficiency	107 (7.2)
Exercise program with resistance/strength component	101 (6.8)
Support services	97 (7)
Physiotherapy or exercise physiologist for exercise prescription	95 (6)
Pharmacist for comprehensive medication review (Home Medication Review item 900)	95 (6)
Occupational therapy for functional/home review	66 (4)

a GP: general practitioner.

b 75+HA: health assessment for those aged 75 years and older.

### PRESS Survey

The PRESS survey was completed by 15 staff (13 nurses, 1 GP, and 1 Practice Manager) from 11 practices (Table 5). All practices had used the FRAIL Scale tool for greater than 6 months prior to survey completion. Free-text comments to the request for other feedback regarding the implementation of the FRAIL Scale tool in the practice are outlined in Multimedia Appendix 3 .

**Table 5.** PRESS survey feedback (n=15) regarding long-term sustainment (continued use) of the FRAIL (Fatigue, Resistance, Ambulation, Illnesses, Loss of weight) Scale rated on a scale from 0 (not at all) to 4 (to a very great extent).

Feedback	Median (IQR)
Staff use the FRAIL Scale tool as much as possible when appropriate	3.0 (2.0‐3.0)
Staff continue to use the FRAIL Scale tool throughout changing circumstances	2.0 (1.0‐3.0)
The FRAIL Scale tool is a routine part of our practice	2.0 (1.5‐4.0)

### Qualitative Interviews

#### Overview

Of the 16 people who consented and were sent the interview questions, 15 took part in the interviews (94% participation rate): 4 practice nurses, 1 GP, and 10 patients (aged 75 years and older). The qualitative results related to the determinants of implementing the two components of the Tool: (1) frailty screening at the time of the 75+HA and (2) risk of frailty/ frailty management.

#### Determinants of Implementing Frailty Screening at the Time of the 75+HA

##### Overview

Qualitative feedback highlighted that the FRAIL Scale Tool focused patient and GP attention on aspects of health and aging. Staff identified that screening at the time of the 75 and over health assessment fit with the general practice workflows, and that the FRAIL Scale tool was easy to use, formalized screening, and allowed tracking of frailty over time. However, the PRESS survey comments suggested key factors would support the adoption of the Tool in the long term.

##### The FRAIL Scale Assessment is Recipient-Centered

Patients reported that they appreciated the opportunity to reflect on their health.

*nobody asks you questions .. like that, usually it’s just whatever you're going to the doctor (for) ….the fact that you know at my age 75…I'm still doing well… So I was kind of quite happy with my assessment.*

[PS14 (Pt)]

*I think it’s great that they do ask those questions because a lot of times you don't mention those sort of things to your doctor when you're there.… those sort of questions .. remind you you're not 21 anymore and your body is not as agile as what it used to be.*

[PS22 (Pt)]

*I probably was pleased with what they went through and thought that the questions give me assurance that I'm doing the right thing.*

[PS15 (Pt)]

*These questions just remind me that …. I have to pay more attention on my … my daily life. And maybe if no one asked me this, maybe something happening that I didn't notice.*

[PS27 (Pt)]

*it’s good learning for me …feeling I have to learn more to keep my body healthy.*

[PS28 (Pt)]

##### Embedding the FRAIL Scale Tool in Practice Processes

GPs and practice nurses reflected on the benefits of having a process for screening for risk of frailty and a method to record and track risk of frailty in the patient chart over time.

*I think.. often there’s an insight to the doctor because we don't always ask those questions.*

[PS17 (GP)]

*they can get an actual number score. Says how frail someone is. If you can look at someone and you can think about their history and everything and they're like, OK, they might be frail. They're becoming a bit frail. But having a number to it makes a difference.*

[PS20 (Nurse)]

The FRAIL Scale tool is easy to use, and although it takes extra time to complete, the Tool improves the care for patients.

*I don't find using the tool complicated at all. I .. it’s easy for us to access. It’s very self-explanatory.*

[PS21 (Nurse)]

*It takes a bit of extra time.. but it means .. we can provide better care for that person… so we have allowed an extra 15 minutes into our appointment.*

[PS20 (Nurse)]

*it’s worthwhile for the government to do this because you get … better outcome(s)..and the frail elderly, definitely you would save a lot more in preventative care.*

[PS17 (GP)]

Staff felt the FRAIL Scale screen fit with the workflows for the 75+HA, and some practices incorporated a link to the Tool from the 75+HA templates in the medical practice software (Best Practice).

*it’s part of the auto fill for the 75+HA. So we do .. daily living and then we go through .. risk factors for osteoporosis. And then falls risk and (the) Frail Scale Tool so it works in perfectly.*

[PS18 (Nurse)]

Introduction of the FRAIL Scale tool, which included management recommendation prompts based on the score in each FRAIL domain, was seen as an advantage by staff.

*much easier because it … gives you prompts … we're actually. .. suggesting actions, whereas before we might have just said that they were frail and .. left it at that, whereas now we're sort of discussing with them .., maybe you should be thinking about doing some balance and falls programs.*

[PS18 (Nurse)]

Some staff found the paper-based FRAIL Scale tool useful as a quick guide to screening and management.

*the frailty management decision tool is quite good.. it’s got the words FRAIL down the side and then it’s got intervention and referral follow up prompts for things that you can do for each section and that’s quite useful. .. easier to look at as an overview.*

[PS18 (Nurse)]

*I do print off recommendations for (management) and (say to the patient) if you want to, you can have a read. You don’t have to. I’m not forcing you to do anything, but this information is here for you when you want it and let us know if you want us to refer (you) to anyone.*

[PS20 (Nurse)]

With the current format of the Tool, documentation and communication within the practice is part of the process of implementing the Tool.

*it’s in the patient notes…… this is what the score number was, .. what it meant. And we also save… the whole thing with the recommendations and everything into their notes as well into correspondence*

[PS20 (Nurse)]

*I do find it is a great resource because..then I'll document it in ..the patient notes. But I will also hand it over then to the GP … they will always see the GP after they see me*

[PS21 (Nurse)]

However, PRESS survey feedback (Multimedia Appendix 3) highlighted that use of the Tool is not business as usual, and building the FRAIL Scale Tool into the practice management software would automate the recording process and make the Tool “more user friendly”.

Patients identified trust in the medical team that facilitated the implementation of screening.

*[Dr] has been my GP for quite some time and she looks after me and my wife really well. So whatever she asks, I'm very happy to discuss with her.*

[PS27 (Pt)]

Some staff felt that completing the risk of frailty screening with robust patients was unnecessary.

*if they're doing.. 3-4 hours of physical activity a week, you possibly don't need to carry on filling the questionnaire.*

[PS21 (Nurse)]

### Determinants of Implementing Risk of Frailty/Frailty Management

#### Overview

While the FRAIL Scale tool raised awareness of the importance of healthy aging, patients identified barriers to accessing management resources. Access to frailty management interventions and resources was limited due to patient motivation and capability related to health, and limited opportunity due to transport issues, cost of resources, or caring duties. Accessing web-based resources was identified as a challenge for those who may not be savvy with technology, and in this case, face-to-face programs were the preferred option. Data recorded by practice staff at the time of frailty screening is presented in Table 6, with further context provided by quotes from the qualitative interviews.

**Table 6.** Barriers to accessing frailty management and resources: quantitative (673 prefrail/frail patients who had a recommendation made) and qualitative (10 patients, 1 general practitioner [GP] interview).

Barriers	Quantitative, n (%)	Qualitative
Health	81 (12)	*I've got blocked arteries in both legs…. I could go out for a walk right now, but I get a certain distance, and I'd have to stop and wait till the… cramps and things in my legs so I can walk home* [PS14 (Pt)]
Transport	81 (12)	*at over 75, .. it's very difficult for them to access the care either because cost or no one's taking them. They don't want to trouble their family, and so they often don't want to follow through* [PS17 (GP)]
Cost	57 (8)	*at over 75, .. it's very difficult for them to access the care either because cost or no one's taking them. They don't want to trouble their family, and so they often don't want to follow through* [PS17 (GP)]
Time	57 (8)	*You gotta have the time and the desire to go walking. So I don't do it too much.* [PS16 (Pt)]
Other (caregiver duties, language, grieving, motivation, technology)	53 (8)	*I've been with the gym for about 15 years, but .. these days I only….go there every once a week … because of my wife, she has dementia... so I don't have much time to do that* [PS27 (Pt)] *I'm here by myself … so you sometimes get a little bit lazy* [PS15 (Pt)] *I haven't been doing them (exercises for shoulder from physio). I've been very lazy, it’s been too cold* [PS19 (Pt)] *another barrier is because we are not IT people. So sometimes ..the programme .. from online .. is a bit difficult for us to join. But if the community can have more practical programmes like face to face .. led by the leader that will be … easy for us to join* [PS28 (Pt)]

The interviews with patients highlighted other determinants of frailty management in primary care that were not identified by practice staff at the time of frailty screening. These included (1) lack of patient knowledge of management options and resources available, (2) social support enhancing participation in physical activity, and (3) location of resources enhancing access.

#### Knowledge of Available Resources

Some patients were unaware of resources available locally or were interested in learning more about healthy lifestyle behaviors.

*They did suggest that maybe that I should … see an exercise Physiologist which .. I've never even heard of before.*

[PS26 (Pt)]

*Teach me … the benefit of .. food and how to eat it better. And how to keep the body .., easier to go to the toilet every day ... That would be helpful.*

[PS28 (Pt)]

#### Relationships and Social Connectedness Were a Facilitator to Exercise

Family, friends, and social interaction were identified as supporting physical activity for several people.

*I go to the gym. I walk with my son every weekend. We have coffee and sometimes, if my daughter can, I walk with her. I see the family all the time. I'm in a good place.*

[PS25 (Pt)]

*My daughter gets onto me about that “do your exercises”, and my other daughter says the same thing.*

[PS19 (Pt)]

*I do Tai chi three times a week…. go to a park with a group… After the Tai Chi we have coffee time and chat.*

[PS28 (Pt)]

#### Access to Resources and Local Programs, and Motivation to Attend

Patients and providers recognized the benefits of local resources for providing access to frailty management.

*In the retirement village where I am, on Tuesday we have exercises, Wednesday we have a bus which takes us shopping, Thursday I play indoor bowls…. And then Friday night I have tea with the neighbours next door…We have a nursing section in the village and they have.. a gym up there*

[PS15 (Pt)]

## Discussion

### Principal Findings

Incorporating the FRAIL Scale tool into the annual health assessment identified 56.5% (839/1484) of the 75 years and over general practice population in this study as frail/at risk of frailty. The predominant frailty management interventions recommended were medication reviews, aged care packages, assessments for depression, and exercise programs. Compared with those who were robust/prefrail, those identified as frail were 4 times more likely to have had a nonelective hospitalization in the 3 months prior to the assessment. Older people living with frailty occupy a large proportion of hospital bed days, at a significant health care cost, and have an increased risk of death [30]. Implementing the FRAIL Scale tool with the annual health assessment provides a funded opportunity to screen for frailty in general practice, prior to onset, and enact prevention/lifestyle interventions to slow or prevent development. Patient feedback highlighted that being involved in the screening process provided an opportunity to reflect on their health, raised awareness of frailty, and prevention and management strategies. Ease of use with screening results linked to actionable management interventions was identified by staff as a valuable component of the FRAIL Scale tool. Patients expressed trust in the primary care team, which may facilitate implementation and highlight the benefits of screening where people receive their usual health care. However, this research has identified individual and health system enablers and barriers experienced by patients to accessing support and services required for effective frailty prevention and management. Determinants of frailty management related to the availability of local resources and the capability, opportunity, and motivation for patients to access these resources. Increasing knowledge for patients and providers on frailty management, providing social support for healthy aging activities, and improving access to management resources were highlighted as potential facilitators to healthy aging.

### Comparison to Prior Work

The rates of prefrailty and frailty (839/1484, 56.5%) in this study are lower than those identified in the pilot study (860/1071, 80%), where both opportunistic screening (at the time of routine procedures) and structured screening were conducted with those aged 75 years and older [11]. This suggests routine screening has the potential for early identification of prefrailty and presents an opportunity for health promotion in early aging.

Other researchers, however, have identified that primary care frailty screening and management tools must have usability and fit with practice workflows and have highlighted the importance of resources or funding for the team to complete frailty assessment [31-34]. This study supports previous findings that implementing the FRAIL Scale tool at the time of the Medicare-funded 75+HA fits with practice workflows, formalizes processes, and renumerates staff time to screen for frailty in this population [11]. In addition, as found in the pilot study, some staff adopted strategies to facilitate the workflow, including incorporating the Tool in the 75+HA templates [11]. However, survey feedback highlighted that embedding the screening tool within practice software was needed to support broader adoption and sustainability of the FRAIL Scale tool in primary care.

In addition, supporting frailty prevention and management for older people in primary care requires knowledge of resources available [15]. While practitioners appreciated the management prompts incorporated in the FRAIL Scale tool, practitioner skill is required to use and encourage behavior change techniques [35] such as social support [36] and goal setting [37] to support healthy aging.

### Strengths

With limited research on prefrailty and frailty in community-dwelling adults in Australia [2,38], this research has highlighted the association of hospitalization with increasing rates of frailty in 2 diverse PHN regions in Australia [39].

The Implementation Research Logic Model [40] has been used throughout the research process to guide planning for the implementation of the FRAIL Scale [41]. The use of the Implementation Research Logic Model has enhanced the implementation process and understanding of implementation determinants framed by CFIR and informed strategies for broader implementation.

### Limitations

First, screening was conducted across only 24 general practices in regions with larger metropolitan populations of older persons and may not be representative of all patients aged 75 years and older. While participation was offered to all practices in each study region, recruitment of metropolitan practices may reflect those practices that had the opportunity to be involved due to adequate staffing, which is often limited in regional, rural, and remote general practices [42]. Preliminary pilot work by the research team in rural and remote Queensland suggests rates of frailty may be higher in nonmetropolitan regions. Second, as risk of frailty screening was completed as part of the 75+HA in primary care settings, the rates of frailty and prefrailty found in this study may not reflect the rates in the broader population of community-dwelling adults aged 75 years or older. Evaluating screening through other avenues, such as community organizations, will be important to reach a broader representation of this age group. Third, the rates of screening for the Aboriginal and Torres Strait Islander population were low in this study. Frailty is known to occur more frequently and at a younger age in the Aboriginal and Torres Strait Islander population [38], and the acceptability of the FRAIL Scale tool to this population was not assessed [38]. Fourth, only 25% (n=15) of staff completed the PRESS survey, with just under 50% (n=11) of practices represented, and qualitative interviews were conducted with only 5 staff. While these results may not reflect all users of the FRAIL Scale, after considering the results from previous interviews with practice nurses and GPs conducted in the pilot study [11] and the free text feedback from the PRESS survey, we did not feel we were gathering any further information and focused on the patient interviews. Finally, while practice staff recorded the management recommendations provided to patients based on the Tool, data was not collected on recommendations adopted by patients following their appointment. In addition, qualitative interviews with patients indicated that social relationships are a facilitator to healthy aging, yet social connectedness was not considered in the FRAIL Scale.

### Future Research

Screening and management with the Tool is currently being evaluated in Western Queensland, where 20% of the population has an Aboriginal or Torres Strait Islander background [43], to understand rates of frailty and the appropriateness of the Tool for other populations and in rural and remote settings. Screening and management recommendations for addressing social connectedness have been incorporated into the Tool for this ongoing research.

While the FRAIL Scale tool can be readily incorporated into the 75+HA, broader implementation requires the Tool to be incorporated into practice software systems. Endorsement of FHIR (Fast Healthcare Interoperability Resources) standards by the Australian Digital Health Agency [44] has provided an opportunity for the research team to develop and evaluate a FHIR-compatible prototype of the FRAIL Scale tool in conjunction with CSIRO researchers [45]. This will improve interoperability across health systems and allow appropriate sharing of data between practice nurses, GPs, and health providers in other health care settings [46].

Importantly, further research is required to understand the effect of screening for risk of frailty in general practice on frailty awareness in the population and whether management recommendations increase adoption of health behaviors to prevent and reverse frailty, encourage proactive self-management, and slow or reverse frailty progression [47]. Consumer engagement will be vital in the development of appropriate, accessible resources for promoting the benefits of frailty prevention and management [7].

While NSPHN and BSPHN are relatively well-resourced regions with accessible programs and support networks, many regions of Australia lack the accessible, coordinated, multidisciplinary workforce required to effectively identify and manage the risk of frailty. This is made more difficult by the poor integration of Commonwealth, state, private, and not-for-profit services [48,49], identified in the aged care sector [50], and exacerbated in rural Australia by a limited local workforce to deliver effective care [42]. Frailty management would benefit from an alliance governance approach, an internationally accepted methodology, effective in binding such diverse health organizations contractually to collectively deliver an agreed program of work [51]. The research team is currently evaluating the implementation of an alliance governance model to support healthy aging in Western Queensland.

### Conclusions

Screening and management of frailty and risk of frailty are not routine in general practice in Australia. A structured approach, using the FRAIL Scale tool at the time of the annual health assessment, has identified 56.5% of the general practice population aged 75 years and older as frail/at risk of frailty. With higher rates of nonelective hospitalization associated with those identified as frail compared with those who were robust/prefrail, routine screening that fits with practice workflows is important in the primary care setting. Importantly, the FRAIL Scale tool provides staff with intervention options to support healthy aging.

## Supplementary material

10.2196/79681 Multimedia Appendix 1 75+ Health Assessment_optimised.

10.2196/79681 Multimedia Appendix 2 Qualitative interview guides_staff and patients.

10.2196/79681 Multimedia Appendix 3 Free text survey responses.

## Abbreviations

75+HA: health assessment for those aged 75 years and older
BSPHN: Brisbane South Primary Health Network
CFIR: Consolidated Framework for Implementation Research
FHIR: Fast Healthcare Interoperability Resources
FRAIL: Fatigue, Resistance, Ambulation, Illnesses, Loss of weight
GP: general practitioner
PHN: primary health network
PRESS: Provider Report of Sustainment Scale
REDCap: Research Electronic Data Capture
SNHN: Sydney North Health Network
