# Supplementary material for: Frailty Screening and Management for Older Australians in General Practice: Mixed Methods Evaluation
Source: Interact J Med Res. 2026 Mar 2;15:e79681. doi: 10.2196/79681 (PMC12954687; doi:10.2196/79681)
Supplement: Multimedia Appendix 1 [file ijmr-v15-e79681-s001.docx]

**75 YEARS + OPTIMISED HEALTH ASSESSMENT**

An assessment of a patient’s health and physical, psychological and social function for the purpose of initiating preventative health care and/or medical interventions as appropriate, may be claimed once every twelve months by an eligible patient.

**Indicate MBS Item Number:** [**<MBS Item Number>**](#BPSFIELD|L|77||||701 : < 30 Minutes|703 : 30-45 Minutes|705 : 45-60 Minutes|707 : > 60 Minutes)

**PATIENT DETAILS**

| Name: <PtFullName> | | | | | DOB: <PtDoB> | |
| --- | --- | --- | --- | --- | --- | --- |
| Gender: <PtSex> | | | | |  | |
| Address:<PtAddress> | | | | | | |
| Telephone: | (home): <PtPhoneH> | | (work): <PtPhoneWk> | | | (mobile): <PtPhoneMob> |
|  | | **Yes=Y, No=N** | | If YES, Language: | | |
| Interpreter Required: | |  | |  | | |

**NEXT OF KIN DETAILS**

| **Name:** <NOKName> | **Address:** | **Phone:** <NOKContact> |
| --- | --- | --- |

**PATIENT CONSENT**

|  | **Yes=Y, No=N** |
| --- | --- |
| Explanation of health check given |  |
| Patient consent for health check was given |  |
| Consent to share/refer to other health providers: |  |
| Comments: | |

**PREVIOUS HEALTH CHECK**

|  | **Yes=Y, No=N** |
| --- | --- |
| Has the patient had a previous health check ?  Date of last health check (if known): |  |
| Previous health check completed by (GP/Practice Name and details): |  |
| Do you currently have a GPMP or TCA ? |  |

**BACKGROUND INFORMATION**

|  | **Comments:** |
| --- | --- |
| Patient says their current health is: |  |
| What are the person's concerns: What matters to you ? |  |
| **Goals for health and wellbeing are:**  **1.**  **2.**  **3.** | |
| Have you seen any other Doctor/GP/Specialist in the last 6 months ? | Name and details: |
| Have you been to hospital / ED in the last 12 months ?  (Check the patient file). | |

**POTENTIALLY PREVENTABLE HOSPITALISATION**

| Is this person at risk of one of the top five conditions for potentially preventable hospitalisation? | | |
| --- | --- | --- |
|  | **Comments** | **Yes=Y, No=N** |
| **COPD**  (if yes, would the person benefit from a referral to a hospital Pulmonary Rehab program?) |  |  |
| **CCF**  (if yes, would the person benefit from a referral to a hospital Heart Failure Rehab program?) |  |  |
| **DIABETES** |  |  |
| **CELLULITIS** |  |  |
| **KIDNEY AND UTIs** |  |  |
| **REFERRALS or ACTIONS REQUIRED:** | | |

**LEGAL ISSUES**

|  | **Comments** | **Yes=Y, No=N** |
| --- | --- | --- |
| Have you made any arrangements for enduring power of attorney should it become necessary? |  |  |
| Have you made any arrangements for guardianship should it become necessary? |  |  |
| If you were ill and unable to make decisions for yourself have you appointed a substitute decision maker or made an Advanced Care Directive ? |  |  |
| **REFERRALS or ACTIONS REQUIRED:** | | |

**OTHER HEALTH CARE PROVIDERS /SERVICES**

| **Do you get regular health care from any other sources ?** | | |
| --- | --- | --- |
|  | **Provider details/ contact** | **Yes=Y, No=N** |
| Audiologist or optometrist |  |  |
| Community nursing |  |  |
| Continence adviser |  |  |
| Dentist |  |  |
| Dietitian |  |  |
| Pharmacist |  |  |
| Physiotherapy |  |  |
| Podiatry |  |  |
| Prosthetist |  |  |
| Psychologist / counsellor |  |  |
| Registered nurse |  |  |
| Social worker |  |  |
| Speech pathologist |  |  |
| Occupational therapist |  |  |
| Other: |  |  |

| **Do you receive any community services ?** | | |
| --- | --- | --- |
|  | **Provider details/ contact** | **Yes=Y, No=N** |
| Home Help - additional paid / unpaid |  |  |
| Home maintenance service |  |  |
| Meals on Wheels or other food  provider service |  |  |
| Daycare |  |  |
| Home care services |  |  |
| Equipment |  |  |
| Local council services |  |  |
| Home modifications |  |  |
| District nurse or other nursing services |  |  |
| Personal care |  |  |
| Community care coordinator |  |  |
| Transport provider  (i.e. Access Cabs, community transport) |  |  |
| Other: |  |  |
| **Do you have ambulance cover ?** | |  |

**MEDICAL HISTORY (Active Items)**

| <CurrentRx> |
| --- |

**RELEVANT FAMILY HISTORY**

| <FamilyHx> |
| --- |

**ALLERGIES (Includes Reactions)**

| <Reactions> |
| --- |

**IMMUNISATION STATUS**

| <Imm> |
| --- |

| Is your patient prepared for winter? |
| --- |

**MEDICATIONS**

|  | **Comments:** | **Yes=Y, No=N** |
| --- | --- | --- |
| Manages own medicines ? |  |  |
| Knowledge of medicines ? |  |  |
| Uses dose administration aid ?  (Webster Pack/Dosette) |  |  |
| Any issues relating to taking medication correctly or regularly ? |  |  |
| Polypharmacy (>5 meds) ? |  |  |
| Checked expiry dates ? |  |  |
| **REFERRALS or ACTIONS REQUIRED:**  **Consider "Home Medicines Review"** | | |

**ALCOHOL**

| <AlcHx> |
| --- |
| Comments: |

**SMOKING**

| <SmkStatus> |
| --- |
| Comments: |

**SOCIAL HISTORY**

|  | **Comments:** | **Yes=Y, No=N** |  |
| --- | --- | --- | --- |
| Who do you live with ?  *Concerns ?* |  |  |  |
| Do you care for someone else ?  If yes, how many hours per day ? |  |  |  |
| Are you cared for by someone else ?  *Concerns ?* |  |  |  |
| What is the current housing situation ?  *Concerns ?* |  |  |  |
| Is there anything you want to do and can't ? |  |  |  |
| What do you do for others ? |  |  |  |
| Have you had any recent stressful events ? |  |  |  |
| Do you have any issues or concerns regarding transport?  If yes, please identify. |  |  |  |
| During the last 4 weeks, was someone available to help if needed / wanted help (For example if you: felt very nervous, lonely or sad, became sick and had to stay in bed, needed someone to talk to, needed help with daily chores, needed help just taking care of yourself) ? |  |  |  |
| Do you participate in religious, social activity or meet regularly with friends or family?  How often? |  |  |  |
| **REFERRAL or ACTIONS REQUIRED:** | | | |

**VISION**

|  | **Comments:** | **Yes=Y, No=N** |
| --- | --- | --- |
| Do you have problems with your eyesight ? |  |  |
| Are you able to read newspapers and books, and watch TV ? |  |  |
| **REFERRAL or ACTIONS REQUIRED:** | | |

**HOME SAFETY & RISK FOR FALLS / INJURIES**

|  | **Comments:** | **Yes=Y, No=N** |  |
| --- | --- | --- | --- |
| Have you had a fall in the past 6 months ? | **If yes, how many ?** |  |  |
| Were you hurt as a result of any of these falls ? |  |  |  |
| What were you doing when you fell ? |  |  |  |
| Did you need assistance to get up from the floor ? |  |  |  |
| Have you had any unsteadiness or 'near-miss falls' in the past 6 months ? |  |  |  |
| Do you have any steps/stairs in your home?  Any difficulties? |  |  |  |
| Do you need any safety equipment (eg: walking aid, home modifications, grab rails fitted)? |  |  |  |
| Is the house free of obvious slipping and/or tripping hazards (e.g. floor mats that slip)? |  |  |  |
| Do you have smoke detectors fitted to your home? (If so, when was the battery last changed ?) |  |  |  |
| **REFERRAL or ACTIONS REQUIRED:** | | | |

**SKIN AND FEET**

|  | **Comments:** | **Yes=Y, No=N** |
| --- | --- | --- |
| Do you have problems with one or both feet? |  |  |
| Are you able to manage your foot and toenail care? |  |  |
| Do you have any areas where your skin is itchy, red, sore, flaky? |  |  |
| Is your skin easily bruised, torn, irritated ? |  |  |
| Last Overall Skin Check ?  (Looking for skin cancers) |  |  |
| **REFERRAL or ACTIONS REQUIRED:** | | |

**FRAILTY SCREEN**

|  |  | **SCALE**  (YES / NO) | **SCORE**  (1 point for "YES") |
| --- | --- | --- | --- |
| **Fatigue** | Do you feel tired most or all of the time? **Intervention:**  **-Consider screening for reversible causes of fatigue**  **-Use k10, Epworth Scale or geriatric depression score during HA**  **Referral:**  **-Geriatrician/specialist for complex care patients**  **-Occupational Therapsit for functional and home review**  **-Psychologist under MHCP**  **-My aged care for loneliness support** |  |  |
| **Resistance** | Can you walk up a flight of stairs without resting? **Intervention:**  **-Consider referring to an individualisd progressive exercise program with resistance & strength**  **Refer:**  **-Physio/EP**  **-Diabetes (group session under Medicare)**  **-Free telephone based health coaching-** [**www.myhealthforlife.com.au**](http://www.myhealthforlife.com.au/) |  |  |
| **Ambulation** | Can you walk around the block unaided? **Intervention:**  **-Consider referring to an individualisd progressive exercise program with resistance & strength**  **Refer:**  **-Physio/Exercise Physiologist**  **-Diabetes (group session under Medicare)**  **-Telephone based health coaching-** [**www.myhealthforlife.com.au**](http://www.myhealthforlife.com.au/) |  |  |
| **Illness** | 5 or more ? **Intervention:**  **- Review indication, side effects and use of medications (evidence for use of some medicines change after 75)**  **-Consider discussing with pharmacist**  **-Consider reducing/de-prescribing superfluous medication**  **Referral:**  **-Pharmacist for comprehensive Medication review (HMR item 900)**  **-Occupational Therapist for functional and home safety review** |  |  |
| **Loss of Weight** | 5% or more in last 12 months **Intervention:**  **-Consider rescreening for reversible causes of weight loss.**  **-Complete mini nutritional assessment in templates**  **-record weight and assess BMI**  **-Consider protein and caloric supplementation/food fortification (75mg protein per day required- range of products available at pharmacy)**  **-Advice and encourage healthy eating; provide Eating well Resource.**  **Referral:**  **-Dietitian for diet review and management**  **-Meal delivery services**  **-Speech Pathology for swallowing review**  **-Dentist for dental review (pain/infection/ill-fitting dentures)**  **-Occupational Therapist for functional and home cooking ability/review** |  |  |
| **Total Score** | 1 point for each "YES" |  |  |
| **Score of 1-2:** at risk of frailty – see frailty management decision tool | | | |
| **Score of 3 or more- FRAIL-** urgently needs referral to reduce further functional decline (e.g. geriatrician, community aged care, exercise physiology, dietitian, pharmacist, other allied health) | | | |
| **REFERRAL or ACTIONS REQUIRED:** | | | |

**NUTRITION**

| **MNA Screening Tool http://www.mna-elderly.com** | | | |
| --- | --- | --- | --- |
|  |  | **SCALE**  (0,1,2 or 3) | **SCORE**  (Add all points) |
| **A.** Has food intake declined over the past 3 months due to loss of appetite, digestive problems, chewing or swallowing difficulties? | 0= severe decrease in food intake  1= moderate in food intake  2= no decrease in food intake |  |  |
| **B.** Weight loss during the last 3 months | 0= weight loss greater than 3 kg  1= does not know  2= weight loss between 1-3kg  3= no weight loss |  |  |
| **C.** Mobility | 0= bed or chair bound  1= able to get out of bed/ chair but does not go out  2= goes out |  |  |
| **D.** Has suffered psychological stress or acute disease in the past 3 months. | 0= Yes  2= No |  |  |
| **E.** Neuropsychological problems | 0= Severe dementia or depression  1= Mild dementia  2= No psychological problems |  |  |
| **F.** BMI | 0= less than 19  1= BMI 19- but less than 21  2= BMI 21 to less than 23  3= BMI 23 or greater |  |  |
| **Total Score** | Screening score (total max 14 pts)  12-14 pts Normal nutritional status  8-11 pts At risk of malnutrition  0-7 pts Malnourished |  |  |
| **Score of 0-7 points:** Refer to dietician or geriatrician/aged care team for full nutritional assessment | | | |
| **Score of 8-11 points:** Address risk factors plus consider adding daily protein supplements (e.g. Hospital strength sustain), and refer for HMR | | | |
| **REFERRAL or ACTIONS REQUIRED:** | | | |

**ORAL HEALTH**

|  | **Comments:** | **Yes=Y, No=N** |
| --- | --- | --- |
| Do you have any of your own natural teeth? |  |  |
| Have you had pain in your mouth while chewing? |  |  |
| Have you lost any fillings, or do you need a dental visit for any other reason ? |  |  |
| Have you avoided laughing or smiling because of problems with your teeth, mouth, or dentures ? |  |  |
| Have you had to interrupt meals because of problems with your teeth, mouth, or dentures ? |  |  |
| Have you had difficulty relaxing or sleeping because of a problem with your teeth, mouth, or dentures ? |  |  |
| **REFERRAL or ACTIONS REQUIRED:**  **Consider "Oral Health for Older People" referral** | | |

**HEARING**

|  | **Comments:** | **Yes=Y, No=N** |
| --- | --- | --- |
| Do you have problems with hearing?  If yes, describe |  |  |
| Are you able to hear and use the telephone? |  |  |
| **REFERRAL or ACTIONS REQUIRED:** | | |

**COGNITION**

|  | **Comments** | **Yes=Y, No=N** |
| --- | --- | --- |
| Tell me how well you think your memory is working these days. |  |  |

**COGNITION SCREEN**

| Perform GPCOG Screening Test | | |
| --- | --- | --- |
| http://gpcog.com.au/  template https://www.alz.org/documents_custom/gpcog(english).pdf | | |
|  | **SCALE**  (Correct / Incorrect) | **SCORE**  (1 point for each correct answer) |
| **Name and Address for subsequent recall test** |  |  |
| **1.** “I am going to give you a name and address. After I have said it, I want you to repeat it. Remember this name and address because I am going to ask you to tell it to me again in a few minutes: John Brown, 42 West Street, Kensington.” (Allow a maximum of 4 attempts). |  | (NO SCORE FOR THIS) |
| **Time Orientation** |  |  |
| **2.** What is the date? (exact only) |  |  |
| **Clock Drawing – use blank page** |  |  |
| **3.** Please mark in all the numbers to indicate the hours of a clock (correct spacing required) |  |  |
| **4.** Please mark in hands to show 10 minutes past eleven o’clock (11.10) |  |  |
| **Information** |  |  |
| **5.** Can you tell me something that happened in the news recently?  (Recently = in the last week. If a general answer is given, eg “war”, “lot of rain”, ask for details. Only specific answer scores) |  |  |
| **Recall** |  |  |
| **6.** What was the name and address I asked you to remember |  |  |
| - John |  |  |
| - Brown |  |  |
| - 42 |  |  |
| - West (St) |  |  |
| - Kensington |  |  |
|  |  |  |
| **Total Score** (score out of 9) |  |  |
| To get a total score, add the number of items answered correctly |  |  |
| **If patient scores 9, no significant cognitive impairment and further testing not necessary.** | | |
| **If patient scores 5-8, more information required. Proceed with Step 2, informant section.** | | |
| **If patient scores 0-4, cognitive impairment is indicated. Conduct standard investigations.** | | |

**GPCOG INFORMANT INTERVIEW (if patient scores 5-8)**

|  | | Date: <TodaysDate> | |
| --- | --- | --- | --- |
| Informant's name: | | | |
| Informant's relationship to patient:  (i.e. Informant is the patient’s) | | | |
| These 6 questions ask how the patient is compared to when s/he was well, say 5-10 years ago.  **Compared to a few years ago:** | | | |
|  | **SCALE**  (YES , NO , Dont know or N/A) | | **SCORE**  (Add all NO,Dont know, N/A answers) |
| Does the patient have more trouble remembering things that have happened recently than s/he used to? |  | |  |
| Does he or she have more trouble recalling conversations a few days later? |  | |  |
| When speaking, does the patient have more difficulty in finding the right word or tend to use the wrong words more often? |  | |  |
| Is the patient less able to manage money and financial affairs (e.g. paying bills, budgeting)? |  | |  |
| Is the patient less able to manage his or her medication independently? |  | |  |
| Does the patient need more assistance with transport (either private or public)?  (If the patient has difficulties due only to physical problems, e.g bad leg, answer is ‘NO’) |  | |  |
| **Total score (out of 6)** |  | |  |
| To get a total score, add the number of items answered ‘no’, ‘don’t know or ‘N/A’ |  | |  |
| **If patient scores 0-3, cognitive impairment is indicated. Conduct standard investigations** | | | |

**CONTINENCE**

|  | **Comments:** | **Yes=Y, No=N** |
| --- | --- | --- |
| Do you ever wet yourself ?  If yes, is this related to coughing and sneezing ? |  |  |
| Do you have any trouble passing urine ?  If yes please describe: |  |  |
| Do you ever lose control of your bowels ? |  |  |
| Have you had a recent change of habit / blood in your motion ? |  |  |
| How often do you go to the toilet at night ? |  |  |
| **REFERRAL or ACTIONS REQUIRED:** | | |

**PERSONAL WELLBEING ASSESSMENT & SAFETY**

|  | **Comments:** | **Yes=Y, No=N** |
| --- | --- | --- |
| During the last 4 weeks, have you been feeling emotional such as anxious, depressed, irritable or downhearted ? |  |  |
| Geriatric Depression Score: |  |  |
| Do you have difficulty sleeping ?  If yes, please describe: |  |  |
| **If patient lives alone ask the following:** |  |  |
| Has a friend or family member made you feel afraid ? |  |  |
| Has a friend or family member hurt you physically ? |  |  |
| Do you need additional support/community services  (e.g.: dressing, bathing, housework, shopping, meals, telephone, garden, other)?  If yes, please identify: |  |  |
| Have you been assessed by the Regional Assessment Service (RAS)?  If yes, date of the assessment  What level of care was deemed appropriate for you at this time? Category - 1 2 3 4  If no, would you like to be assessed? |  |  |
| **REFERRAL or ACTIONS REQUIRED:** | | |

**PHYSICAL ASSESSMENT**

|  | **Measurements** | **Any Changes ?** |
| --- | --- | --- |
| **Height:** |  |  |
| **Weight:** |  |  |
| **Waist:** |  |  |
| **BMI:** |  |  |
| **BP:** |  |  |
| **Pulse:** |  |  |
| **REFERRAL or ACTIONS REQUIRED:** | | |

**MOBILITY / ACTIVITY**

|  | **Comments:** | **Yes=Y, No=N** |
| --- | --- | --- |
| Do you use a walking aid, such as a walking stick, frame, wheelchair or motorised scooter? |  |  |
| Can you carry objects (e.g.: meals easily and safely from the kitchen to your dining area)? |  |  |
| Are you able to look after the inside/outside of the house? |  |  |
| Do you have difficulty gripping utensils or handrails? |  |  |
| **Perform TUG test**: Timed Up and Go _________ seconds (> or = 14 secs high risk falls)  https://www.unmc.edu/media/intmed/geriatrics/nebgec/pdf/frailelderlyjuly09/toolkits/timedupandgo_w_norms.pdf | | |
| **Perform 4 Metre Walk Test** _________ seconds (< 5 secs Normal- > 5 secs requires further investigation (Frailty)  https://www2.gov.bc.ca/assets/gov/health/practitioner-pro/bc-guidelines/frailty-gaitspeed.pdf | | |
| **Check Grip strength** >30 kg for males >25kg for females in dominant hands | | |
| **REFERRAL or ACTIONS REQUIRED:** | | |

**GP / Nurse: _________________**

**SIGNATURE:______________**

**DATE: <TodaysDate>**

**This section may be completed by the GP**

**MEDICAL EXAMINATION**

|  | **Measurements** | **Comments** |
| --- | --- | --- |
| **Cardiovascular:** |  |  |
| **Respiratory:** |  |  |
| **Neurological:** |  |  |
| **Gastrointestinal:** |  |  |
| **Other as appropriate:** |  |  |
| **REFERRAL or ACTIONS REQUIRED:** | | |

**INVESTIGATIONS**

|  | **Measurements** | **Comments** |
| --- | --- | --- |
| **BGL:** |  |  |
| **Urinalysis:** |  |  |
| **Last bone density?** | **date:** |  |
| **Other tests as appropriate e.g. blood levels for medications, electrolytes, kidney health check (creatinine, eGFR, urine ACR + BP), spirometry, ECG:** |  |  |
| **REFERRAL or ACTIONS REQUIRED:** | | |

**SUMMARY OF HEALTH ASSESSMENT**

**Patient Name: <PtFullName>** DOB: <PtDoB>

| Based on consideration of evidence from patient history, examinations, lifestyle risk factors and results of any investigation. | |
| --- | --- |
| Patient’s overall health is:  (stable/deteriorating) |  |
| Existing health problems: (List) |  |
| Identified risk factors: (List) |  |
| Health advice/recommendations provided to patient/carer: |  |
| Any additions to the social history required? |  |

**INTERVENTION**

Please check HealthPathway referral information for your area: **www.healthpathways.org.au**

Sydney: **sydney.healthpathways.org.au**

South Eastern Sydney: **coming soon**

| **I believe the patient would benefit from:** | | |
| --- | --- | --- |
|  |  | **Yes=Y, No=N** |
| GPMP | MBS Item No 721 |  |
| GPMHP | MBS Item No 2710 |  |
| TCA | MBS Item No 723 |  |
| Chiropractor |  |  |
| Diabetes education |  |  |
| Dietician |  |  |
| Exercise Physiologist |  |  |
| Hearing |  |  |
| Optometrist |  |  |
| Ophthalmologist |  |  |
| Occupational Therapist |  |  |
| Physiotherapy |  |  |
| Podiatry |  |  |
| Specialist |  |  |
| Speech Pathology |  |  |
| Social Work |  |  |
| Home Medicines Review | MBS Item No 900 |  |
|  | MBS Item No 735-758 |  |
| "My Aged Care Referral" |  |  |
| Carer Support |  |  |
| Community Health |  |  |
| Day Therapy/ Day Care |  |  |
| Diabetes Education |  |  |
| Dental |  |  |
| Falls Preventation |  |  |
| FRAIL Assessment |  |  |
| Geriatrician Review |  |  |
| Home care services |  |  |
| Incontinence Nurse / Stomal Therapist |  |  |
| Meals on Wheels or similar |  |  |
| Respite services |  |  |
| Social links |  |  |
| Other |  |  |
| **Comments:** | | |

**ACTION PLAN**

| Identified PERSONAL GOALS: **What Matters to You?** and **PLAN of ACTION** |
| --- |

**Nurse:__________________________ Signature:________________________**

**GP DETAILS**

| Details of person completing this assessment:  Name: <DrName>  Practice: <Practice> Phone: <UsrPhone>  Fax: <UsrFax> Provider No.: <DrProviderNo>  Signature:____________________________  Date: <TodaysDate> |
| --- |

**Review for next Health Assessment - Date:**
