# Supplementary material for: Frailty Screening and Management for Older Australians in General Practice: Mixed Methods Evaluation
Source: Interact J Med Res. 2026 Mar 2;15:e79681. doi: 10.2196/79681 (PMC12954687; doi:10.2196/79681)
Supplement: Multimedia Appendix 2 [file ijmr-v15-e79681-s002.docx]

**Interview Guide for Health Professional**

**Identifying the enablers and barriers to using the FRAIL Scale Tool**

**Global Question**

1. I’d like to start by hearing your general thoughts about the FRAIL Scale Tool and your experience using it?

**Intervention characteristics**

1. How complicated is the FRAIL Scale Tool?
2. What other methods do you currently use to assess frailty in your practice?

How does the FRAIL Scale Tool compare to other methods?

1. How well does the FRAIL Scale Tool fit with existing work processes and practices in your setting?
2. Has the FRAIL Scale Tool replaced or does it compliment a current program or process? In what ways?
3. What kind of training, information & materials have you received regarding the FRAIL Scale Tool? Are there any other types of information or resources that would have been helpful to support you when using the app or for the patients?

**Implementation Climate**

1. What is the general level of receptivity in your organization to implementing the FRAIL Scale Tool?
2. To what extent has your organization/unit set goals for implementing the FRAIL Scale Tool?

**Patient Needs & Resources**

1. How well do you think the FRAIL Scale Tool meets the needs of the patients?
2. How have your patients responded to the FRAIL Scale Tool?
3. Are there some patients that the tool is more suitable for/not suitable for and why?
4. How have your patients responded to the recommendations you are making after you have done the assessment with the Tool?
5. Are there some patients that these recommendations are more suitable for/not suitable for and why?
6. What programmes (e.g. healthy lifestyle/ activity) and providers (AHP/ geriatricians) are available to refer patients to?
7. How have you and patients found accessing the resources and referral options? Are there any barriers?

**Close** Finally, do you have anything else you’d like to add?

**Interview Guide for Patients**

**Identifying the enablers and barriers to using the FRAIL Scale Tool**

**Interviewer Introduction**

Thank you for agreeing to take part, we really appreciate you taking the time to tell us your thoughts about the FRAIL Scale Tool.

As explained on the Information Sheet, I’ll be recording the interview so I have an accurate record of what was said, but the recording and the transcript will be de-identified and no name linked data will be reported.

Do you have any questions you’d like to ask before I start recording?

During your over 75 health assessment you were asked 5 questions to assess your risk of frailty. These questions asked if you were **1) Feeling fatigued most or all of the time; 2) Had difficulty walking up 10 steps without resting; 3) Had difficulty walking 300metres or around the block; 4) had 5 or more illnesses and; 5) lost >5% weight in the last 12 months**

1. I’d like to start by hearing your general thoughts about the FRAIL Scale questions?
2. How do the FRAIL Scale questions compare to other questions asked in your health assessment? How do you feel the FRAIL Scale questions fit with the 75 and over health assessment.

Was the meaning of the questions clear?

1. How do you feel about being asked the FRAIL Scale questions?
   1. Did you have any feelings of anticipation? Stress? Enthusiasm? Why?
2. Who do you feel is the best person in the practice to ask the FRAIL Scale questions?
3. What kind of information did you learn from being asked the FRAIL Scale questions?
4. What kinds of changes will you make from being asked the FRAIL Scale questions?
5. What programmes (e.g. healthy lifestyle/ activity) and providers (allied health professionals such as exercise physiologist/ geriatricians) were you referred to?
6. Do you feel these programmes and providers are accessible? Are there any barriers to accessing these programs/providers?
7. What type of programmes and resources would you prefer?
8. Finally, do you have anything else you’d like to add?
9. Would be interested in being a consumer representative for this research program?
