# Supplementary material for: Frailty Screening and Management for Older Australians in General Practice: Mixed Methods Evaluation
Source: Interact J Med Res. 2026 Mar 2;15:e79681. doi: 10.2196/79681 (PMC12954687; doi:10.2196/79681)
Supplement: Multimedia Appendix 3 [file ijmr-v15-e79681-s003.docx]

Additional File 3**:** Free text comments to the survey question

| ***Do you have any other feedback regarding the implementation of the FRAIL Scale tool in your practice?*** |
| --- |
| “It’s a useful tool” |
| “Usually completed as part of our 75+ health check”  “Embedded in our 75+HAs. Would like to use it when we see patients for wound care as well. Will put it in our wound care template going forward.” |
| “Essentially, we have been covering these questions at our 75+HA. The addition of the FRAIL scale tool provides a more digestible framework. Prior to participation in the study all elements of the FRAIL scale tool were assessed individually. The FRAIL scale tool provided improved framework of the assessment. Happy to continue this practice.” |
| “It was a very helpful tool but once the researched has finished, we also stopped doing it. We just used it to help for research purposes but most doctors are happy to use it as their reference.”  “Really should utilise it more. Factors affecting this are time constraints, and if it was integrated in the clinical software. It would be of more use if it was integrated into the practice software.”  “I was only using it as part of the yearly health assessment and am generally pushed for time.” |
| “Wishing the PMS *(practice management software)* would allow recording as an observation” |
| “The FRAIL scale is on the desktop, but it meant a few more clicks to open it and I got out of the habit of using it. Being accessible on BP *(Practice Software)* under the 'Clinical' section, where we can easily access percentile charts, mini mental, diabetes risk assessment etc would make the FRAIL scale more user friendly.” |
